# Hypoglycaemic and Antioxidant Effects of Propolis of Chihuahua in a Model of Experimental Diabetes

**DOI:** 10.1155/2018/4360356

**Published:** 2018-03-11

**Authors:** Nelly Rivera-Yañez, Mario Rodriguez-Canales, Oscar Nieto-Yañez, Manuel Jimenez-Estrada, Maximiliano Ibarra-Barajas, M. M. Canales-Martinez, M. A. Rodriguez-Monroy

**Affiliations:** ^1^Lab. Inmunobiología (L-321), UNAM FES Iztacala, Avenida de los Barrios Número 1, Colonia Los Reyes Iztacala, 54090 Tlalnepantla, MEX, Mexico; ^2^IPN, Escuela Nacional de Ciencias Biológicas, Av. Wilfrido Massieu, Gustavo A Madero, 07738 Ciudad de México, Mexico; ^3^Instituto de Química, Universidad Nacional Autónoma de México, Circuito Exterior, Ciudad Universitaria, 04510 Coyoacán, DF, Mexico; ^4^UBIMED, UNAM FES Iztacala, Avenida de los Barrios Número 1, Colonia Los Reyes Iztacala, 54090 Tlalnepantla, MEX, Mexico; ^5^Lab. Farmacognosia, UBIPRO, UNAM FES Iztacala, Carrera Biología, Tlalnepantla, MEX, Mexico

## Abstract

Propolis is a bee-collected natural product that has been proven to have various bioactivities. This study tested the effects of a Mexican propolis on streptozotocin-induced diabetes mellitus in a murine model. The results showed that an ethanolic extract of propolis of Chihuahua (EEPCh) significantly inhibited increases in blood glucose and the loss of body weight in diabetic mice. EEPCh increased plasma insulin levels in STZ-diabetic mice, whereas, in untreated diabetic mice, there was no detection of insulin. EEPCh had a high antioxidant capacity (SA_50_ = 15.75 *μ*g/mL), which was directly related to the concentrations of total phenols (314 mg GAE/g of extract) and flavonoids (6.25 mg QE/g of extract). In addition, increased activities of the enzymes superoxide dismutase, catalase, and glutathione peroxidase were observed in diabetic mice treated with EEPCh. Compounds such as pinocembrin, quercetin, naringin, naringenin, kaempferol, acacetin, luteolin, and chrysin were identified by HPLC-MS analysis. This investigation demonstrated that propolis of Chihuahua possesses hypoglycaemic and antioxidant activities and can alleviate symptoms of diabetes mellitus in mice. These effects may be directly related to the chemical composition of propolis, as most of the compounds identified in propolis are reportedly active in terms of the different parameters evaluated in this work.

## 1. Introduction

Propolis is a resinous, complex apiarian product that is collected and produced by honey bees from different floral substances, beeswax, and salivary secretions [1]. Propolis is characterized by its contents of resins (approximately 50%), wax (30%), essential oils (10%), pollen (5%), and other organic components (5%) [2]. Importantly, each propolis varies in composition depending on its geographical area of origin and the type of flora in that region [3]. Notably, propolis possesses a broad spectrum of biological activities, including antimicrobial, anti-inflammatory, antiviral, cytotoxic, antioxidant, and immunomodulatory [4, 5]. In addition, recent studies have shown that propolis has hypoglycaemic effects and antioxidant capacity [6, 7].

Diabetes is a chronic metabolic disorder characterized by hyperglycaemia that presents a major worldwide health problem [1]. In view of its epidemic proportions, diabetes stands out as one of the most urgent medical problems of the 21st century [8]. According to the World Health Organization (WHO), in the year 2000, the prevalence of diabetes mellitus was 171,000,000, and it is estimated that the prevalence of this disease will reach 439,000,000 by 2030 [9]. Diabetes mellitus is characterized by absolute or relative deficiencies in insulin secretion and/or insulin action associated with chronic hyperglycaemia and disturbances of carbohydrate, lipid, and protein metabolism [8].

Hyperglycaemia is an important factor responsible for intense oxidative stress in diabetes, and the toxicity induced by glucose autoxidation is likely to be one of the important sources of reactive oxygen species. Several intra- and extracellular antioxidant defence mechanisms counteract the destructive effects of free radicals by attenuating or inhibiting their activities. However, in diabetes mellitus, the oxidative stress exceeds the antioxidant defence mechanisms of the body [7].

Diabetes is associated with the generation of reactive oxygen species (ROS), which cause oxidative damage to the heart, kidneys, eyes, nerves, liver, small and large blood vessels, and the gastrointestinal system [8].

As a result of the plethora of scientific evidence proposing the involvement of oxidative stress in the pathogenesis of diabetes and its complications, interest has grown in the use of natural antioxidants as a new strategy for alleviating the oxidative damage associated with diabetes [9]. Therefore, the present study aimed to investigate the hypoglycaemic and antioxidant activities of EEPCh in a model of experimental diabetes.

## 2. Material and Methods

### 2.1. Chemicals

Streptozotocin (STZ) was purchased from Sigma-Aldrich (St. Louis, USA) and stored at −20°C. Rat/mouse insulin ELISA kits were obtained from EMD Millipore. Aluminium chloride (AlCl_3_), 2,2-diphenyl-1-picryl-hydrazyl (DPPH), Folin–Ciocalteu reagent, sodium carbonate, and ethanol were purchased from Sigma-Aldrich (St. Louis, MO, USA). Authentic flavonoid standards (vanillin, quercetin, catechin, luteolin, pinocembrin, chrysin, baicalein, myricetin, naringenin, naringin, gallic acid, catechol, apigenin, acacetin, and kaempferol) were purchased from Sigma-Aldrich (St. Louis, MO, USA).

### 2.2. Biological Materials

Propolis samples were collected by Ing. Martín Balcorta Baeza in November, 2014, from the apiary located in Ejido Concordia, Aquiles Serdán municipality, Chihuahua, Chihuahua, Mexico.

### 2.3. Preparation of the Ethanolic Extract of Propolis of Chihuahua (EEPCh)

The propolis extract was obtained by maceration at room temperature. Propolis (300 g) was placed in a flask containing 1 L of 70% ethanol. Next, the solution was filtered and then distilled under reduced pressure in a rotary evaporator [10]. The extract was placed in glass containers until evaporation of the solvent was complete, thus yielding the ethanol extract of propolis of Chihuahua (EEPCh). The yield of EEPCh was 202.45 g (67.48%).

### 2.4. Experimental Animals

Healthy male adult CD1 mice (seven weeks old) were used. The animals were cared for according to the guidelines of the Federal Regulations for Animal Experimentation and Care (NOM-062-ZOO-1999, Ministry of Agriculture, Mexico), which were approved by the Institutional Ethics Committee of the Universidad Nacional Autónoma de Mexico, Facultad de Estudios Superiores Iztacala. All animals were housed with six mice per cage and were fed with a standard pellet diet and were allowed free access to water. The animals were maintained at a constant temperature (22 ± 2°C) and 50 ± 5% relative humidity with a 12 h light–dark cycle.

### 2.5. Induction of Experimental Diabetes

Diabetes was induced by intraperitoneal injection (i.p.) of a single dose of 130 mg/kg body weight of streptozotocin (STZ). STZ was dissolved in freshly prepared 0.05 M citrate buffer (pH 4.5). After 72 h, all mice were fasted for 4 h and their blood glucose levels were monitored from the tip of the tail vein using a glucose kit and an autoanalyser (ACCU-CHEK Active, Roche diagnostics). The mice used in the experiments were considered diabetic when their fasting blood glucose levels were above 250 mg/dL [11].

### 2.6. Experimental Groups and Treatment

Two batches (groups) of mice were used, one for immunohistochemistry and the other to obtain the homogenates of the pancreatic tissue.

The experimental animals were randomly assigned into 3 groups of six animals each and received the following treatments: Group I, healthy (H); Group II, diabetics (D); and Group III, diabetics treated with propolis (D/P) 0.3 g/kg/day of propolis [7]. The freshly prepared extracts of propolis were orally administered one week after the mice were considered as diabetics (glucose levels greater than 250 mg/dL). The propolis administration was daily for 15 days. Body weights and blood glucose levels were measured at 3-day intervals after fasting for 4 h.

### 2.7. Effects of EEPCh on Blood Glucose Levels and Body Weight in Experimental Groups

During the study period of 15 days, the blood glucose levels of the mice were recorded every 3 days. Blood was extracted from the tip of the tail vein after fasting for 4 h, and the blood glucose levels were determined using a glucometer. The body weights of mice were also recorded at the same times using an electronic balance (ADAM, 120 g × 0.001 g, USA).

### 2.8. Effect of EEPCh on Serum Insulin Levels

Blood was collected at the end of treatment (day 15) in tubes without heparin or EDTA and centrifuged at 10,000 rpm for 5 min. The serum insulin levels were determined using a mouse insulin ELISA kit (EMD Millipore, rat/mouse insulin ELISA kit, USA), which quantifies insulin using a sandwich-technique enzyme immunoassay. After incubation, the plate was read at 450 nm in a Bio-Tek EL800 plate reader (Bio-Tek). The intensity of the colour generated was directly proportional to the amount of insulin in the sample.

### 2.9. Effects of EEPCh on Pancreatic Islets

At the end of the experimental period, the animals were sacrificed and each pancreas was quickly removed. An incision was made in the abdomen and the liver was lifted to allow for observation of the bile duct, through which 1 mL of Bouin fixative was injected. The pancreas was then obtained by carefully cutting along the borders of the pancreas to separate it from the organs to which it was adhered. The samples were fixed for 24 h at 4°C, the tissues were dehydrated using a graded alcohol series, and finally, the samples were embedded in paraffin (Paraplast, Merck Millipore, USA) following routine histological techniques. Each pancreas was cut to 4 microns of thickness on a rotary microtome (Leica, RM2125RT, Germany). Immunohistochemistry using an insulin antibody (H-86, sc-9168; Santa Cruz Biotechnology, Inc.) was performed to detect insulin in the pancreatic islets of each experimental group, and the slides were examined under a light microscope (Motic, BA310E, China) at 40x magnification.

### 2.10. Antioxidant Capacity of EEPCh

The antioxidant capacity of the propolis extract was measured using a DPPH assay according to a previously described procedure [12]. In an ELISA plate, 50 *μ*L of propolis extract was added at different concentrations (1–100 *μ*g/mL) in triplicate. DPPH solution (150 *μ*L) was added and the plate was then shaken and kept in the dark at room temperature for 30 min at 37°C. The absorbance was measured at 540 nm using a Bio-Tek EL800 plate reader (Bio-Tek). LC-MS grade methanol was used as a blank sample, and a DPPH solution (100 *μ*M) was used as the control. Quercetin was used as the reference (positive control) under the same conditions as the problem solution. The antioxidant capacity was determined according to the following equation:

(1) %  reduction=absorbance  of  control−absorbance  of  sampleabsorbance  of  control∗100.

The concentration with 50% antioxidant capacity (SA_50_) was determined graphically.

### 2.11. Total Phenolic Content of EEPCh

The total phenolic content (TPC) of the propolis extract was determined by Folin–Ciocalteu reagent [13]. The sample was tested in triplicate and the absorbance of the resulting blue coloured solution was measured at 760 nm using a UV–Vis spectrophotometer (DU 640 Spectrophotometer, Beckman, Brea). The total phenolic content was estimated using a calibration curve generated using serial concentrations of gallic acid (0.00625, 0.0125, 0.025, 0.05, 0.1, and 0.2 mg/mL), and total phenolics were expressed as milligrams of gallic acid equivalent per gram of extract (mg of GAE/g of extract).

### 2.12. Total Flavonoid Content of EEPCh

The total flavonoid content (TFC) of the propolis extract was estimated using a previously described colorimetric method based on the formation of an aluminium chloride complex [13]. In an ELISA plate, 200 *μ*L of the mixture was added in triplicate and the samples were incubated for 10 min in the dark at room temperature. A calibration curve was generated using different concentrations (1–100 *μ*g/mL) of quercetin. The absorbance was read at 415 nm in a Bio-Tek EL800 plate reader (Bio-Tek) and the total flavonoid content was expressed as milligrams of quercetin equivalent per gram of extract (mg of QE/g of extract).

### 2.13. Effects of EEPCh on Some Parameters of Oxidative Stress

At the end of the experimental period, the animals were sacrificed, each pancreas was quickly removed, and the pancreatic tissues were homogenized (100–300 mg) using a Bullet Blender (Next Advance, Inc. USA). The homogenate was centrifuged at 10,000 rpm for 5 min and the collected supernatant was stored at −80°C. The enzymatic activities of superoxide dismutase (SOD), catalase (CAT), and glutathione peroxidase (GPx) were all determined using the methods provided by the assay kits (Cayman Chemical Company, USA).

### 2.14. Analysis of the Chemical Composition of EEPCh by High-Performance Liquid Chromatography (HPLC-DAD)

HPLC was used to chemically characterize 30 *μ*L of the propolis extract. The extract was injected at a concentration of 3 mg/mL into a Hewlett-Packard HP model 1100 series (Hewlett-Packard, Wilmington DE, USA) HPLC equipped with a diode array detector (DAD) 1100 operated with ChemStation A0903 under the following parameters: separation isocratic using a mobile phase, methanol : acetonitrile : water (25 : 25 : 50) acidified with formic acid (1%) for 60 minutes; column, Discovery C-18 (250 × 4.6 mm), at 269 bar pressure and a temperature range of 22°C-23°C; flow rate, 1 mL/min; detector array of diodes with detector setting at 260 nm; and full scanning of 200–400 nm. The constituents were identified based on a comparison of the retention time and UV spectrum with those of the standards.

### 2.15. Analysis of the Chemical Composition of EEPCh by Liquid Chromatography–Mass Spectrometry (HPLC-MS)

HPLC-MS analysis was performed using an Agilent 1200 Infinity LC coupled to an Agilent 6230 TOF with an Agilent Dual ESI Source (ESI SG14289023) and Mass Hunter Wokstation Software, Version B.05.01, Build 5.01.5125.3 operating in the negative ionization mode. Capillary voltage was 4000 V; dry gas temperature was 250°C; nitrogen was used as the dry gas at a flow rate 6 L/min; nebulizer pressure was 60 psi; fragmentor was 200 V; MS range was 50–1300 *m*/*z*; MS acquisition rate was 1 spectrum/s.

The chromatographic separation was accomplished using a HPLC (Infinity Series 1200, Agilent Technologiest, Germany) equipped with a Kinetex 2.6 u, C1800A column (150 × 2.1 mm) (Phenomenex, USA). The column temperature was maintained at 25°C. The following gradient program was used, along with a mobile phase consisting of water :  acetonitrile (90 : 10) with 0.1% formic acid (solvent A) and methanol : acetonitrile (90 : 10) with 0.1% formic acid (solvent B). This initial term for 3 min in an isocratic elution is composed of 100% solvent A followed by 3–11 min: 65% A-35% B; 11–20 min: 55% A-45% B; 20–35 min: 100% B; and 25 min: 100% B, v/v. The flow rate was 0.2 mL/min, and the injection volume was 20 *μ*L (3 mg/mL).

### 2.16. Statistical Analysis

All data concerning hypoglycaemic activity, body weight, detection of insulin,* in vitro* antioxidant capacity, total phenolic content, total flavonoids content, and the antioxidant enzymes activity (SOD, CAT and GPx) were expressed as the means ± SD. Statistical differences between the treatments and the controls were tested by one-way analysis of variance (ANOVA) using the GraphPad-Prism (version 6.0) statistical analysis software. A difference in the mean values of *P* < 0.05 was considered statistically significant.

## 3. Results

### 3.1. Effects of EEPCh on Blood Glucose Levels and Body Weight

**Figure 1:**
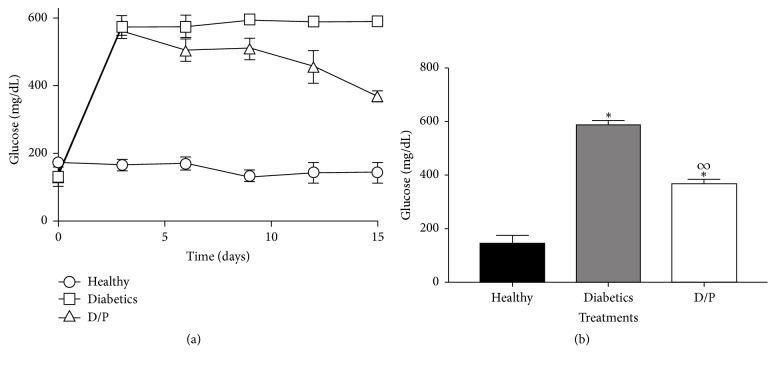
Effect of propolis on blood glucose levels. Results are from 15 days of treatment. Diabetic mice treated daily with propolis (300 mg/kg) have decreased glucose levels compared with the untreated diabetic group (Figures 1(a) and 1(b)). The values represent the means of *n* = 6. Significant differences were determined with multiple *t*-tests, *P* < 0.05. *∗* indicates statistically significant differences with respect to the healthy group. *∞* indicates statistically significant differences with respect to the diabetic group.

During the treatment period, diabetic mice showed constant hyperglycaemia from day 3 until day 15, whereas the group of diabetic mice treated with propolis showed significantly decreased glucose levels from day 9 until the end of the treatment period. In the healthy mice, constant glucose levels were maintained below 200 mg/dL (Figure 1).

It is important to note that the dose used (0.3 g/Kg/day of propolis) is not toxic, according to the OECD 423 acute toxicity test [14] (data not shown).

### 3.2. Body Weight Monitoring

**Figure 2:**
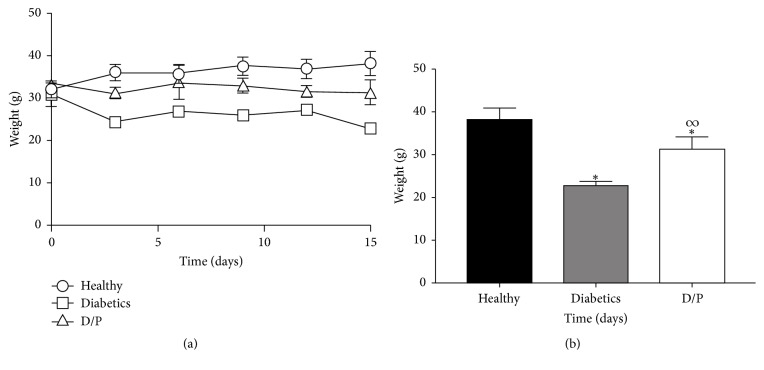
Effect of propolis on body weight of mice. Results from 15 days of treatment are shown. Unlike the group of diabetic mice without treatment, the diabetic mice treated with propolis daily (300 mg/kg) showed no drastic weight loss (Figures 2(a) and 2(b)). The values represent the means of *n* = 6. Significant differences were determined with multiple *t*-tests, *P* < 0.05. *∗* indicates statistically significant differences with respect to the healthy group. *∞* indicates statistically significant differences with respect to the diabetic group.

The administration of propolis during the 15 days of treatment showed a considerable effect on the weight loss of the diabetic mice. As shown in Figure 2(a), the mice that were given propolis showed significantly greater body weights of above 30 g compared to the diabetic mice, which weighed below 30 g; the healthy mice maintained the highest weight at a constant 35 g. At the end of treatment (Figure 2(b)), the weights of the diabetic mice treated with propolis (D/P) were significantly different compared to those of the diabetic mice.

### 3.3. Effects of EEPCh on Serum Insulin Levels

**Figure 3:**
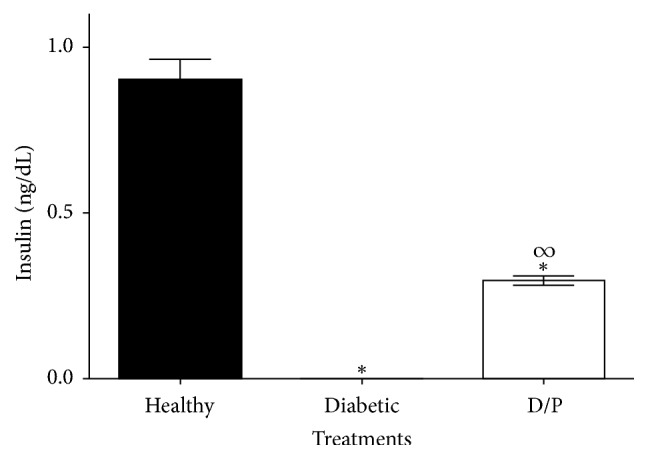
Serum insulin levels on day 15 of treatment. Serum insulin was detected (0.3 ± 0.02 ng/mL) in diabetic mice treated daily with propolis (300 mg/kg), whereas untreated mice showed no insulin levels. The values represent the means of *n* = 6. Significant differences were determined by ANOVA. *∗* indicates significant differences with respect to the healthy group. *∞* indicates significant differences with respect to the diabetic group (*P* < 0.05).

Since a decrease in blood glucose levels was found, the insulin levels were then determined in the experimental groups. At the end of treatment (day 15), the insulin levels (expressed in ng/mL) in the group of healthy mice (H) showed a value of 0.9 ng/mL, whereas in the group of diabetic mice the presence of insulin was not detected. In the group of mice treated with propolis, insulin levels of 0.3 ng/mL (Figure 3) were recorded, with significant differences compared with those of the diabetic mice (D).

### 3.4. Effects of EEPCh on Pancreatic Islets

**Figure 4:**
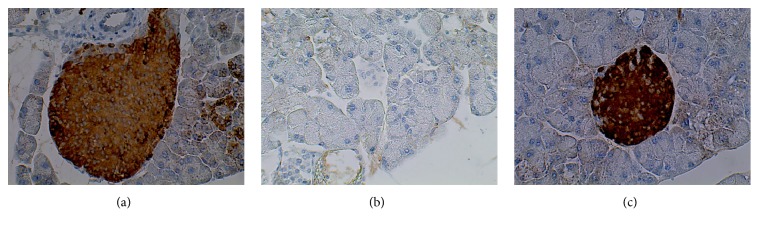
Immunohistochemical detection of pancreatic insulin in the different experimental groups. Healthy mice showed insulin production (Figure 4(a)), whereas untreated diabetic mice show no insulin production (Figure 4(b)). The oral administration of propolis to diabetic mice favours the production of insulin in the pancreatic islets (Figure 4(c)). Each image is representative of 6 independent experiments. Microphotographs (40x).

After the oral administration of propolis, differences in the size of the islets were observed. Thus, immunohistochemistry was performed to determine whether the islets were still producing insulin. In this assay, it was found that the islets in the group of healthy mice (H) and the diabetic group treated with propolis (D/P) contained insulin, whereas the islets in the group of diabetic mice (D) did not contain insulin (Figures 4(a), 4(b), and 4(c)).

### 3.5. Antioxidant Capacity (SA_50_), Total Phenolic Content (TPC), and Total Flavonoid Content (TFC) of EEPCh

The EEPCh showed a SA_50_ = 15.75 *μ*g/mL. According to the literature, phenols are primarily responsible for antioxidant capacity. The total phenolic content of EEPCh was 314 mg GAE/g of extract, and the total flavonoid content was 6.25 mg QE/g of extract.

### 3.6. Effects of EEPCh on Some Parameters of Oxidative Stress (SOD, CAT, and GPx)

**Figure 5:**
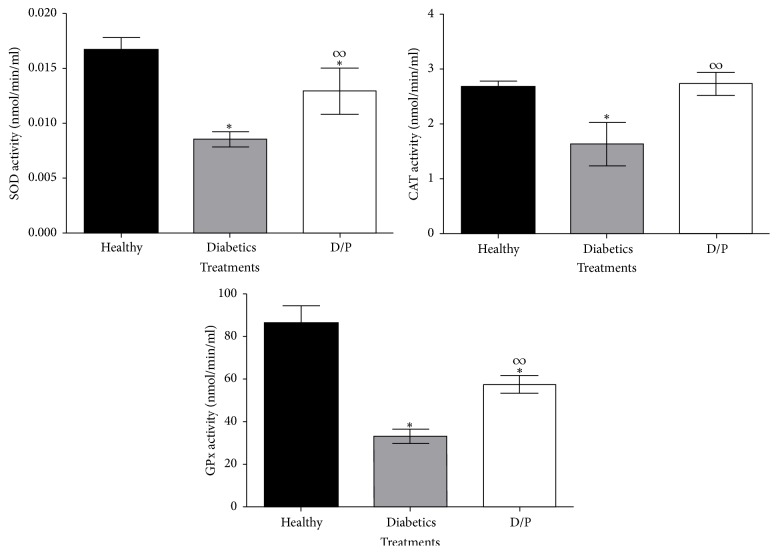
Effects of EEPCh on the enzymatic activities SOD, CAT, and GPx. Diabetic mice treated with propolis (D/P) showed increased activities of antioxidant enzymes compared with those of untreated mice. Notably, in the treated mice, the CAT activity shows no differences relative to that of healthy mice. The values represent the means of *n* = 6. Significant differences were determined by ANOVA. *∗* indicates significant differences with respect to the healthy group. *∞* indicates significant differences with respect to the diabetic group (*P* < 0.05).

High concentrations of glucose in diabetes lead to oxidative stress by increasing the levels of reactive oxygen species (ROS) and decreasing the antioxidant defences of the organism. Therefore, the effects of propolis on the enzymatic activities of enzymes involved in the antioxidant system (SOD, CAT, and GPx) were determined at the end of treatment (day 15) with propolis (Figure 5) using colorimetric methods. As shown in Figure 5, the activities of these three enzymes were diminished in the diabetic mice. Nevertheless, the mice treated with propolis showed increased enzymatic activities of SOD, CAT, and GPx. This is an important result that indicates a reduction in oxidative stress in the D/P group compared with the D group.

### 3.7. Analysis of the Chemical Composition of EEPCh by HPLC-DAD and HPLC-MS

The compounds in the sample were identified according to their absorption maxima under low ultraviolet light (*λ*_max⁡_), their retention times, and HPLC-MS. In total, 8 compounds were identified (Table 1).

## 4. Discussion

Since ancient times, propolis has been used extensively by humans for its beneficial effects and to treat many diseases and conditions. Propolis is a natural remedy and a popular alternative medicine for various diseases. Current applications of propolis include formulations for cold syndromes (upper respiratory tract infections, common cold, and flu-like infections), and dermatological preparations are useful in wound healing and in treating burns, acne, herpes simplex and genitalis, and neurodermatitis [15]. Propolis is known to have antibacterial, antifungal, antioxidant, anti-inflammatory, antiviral, immunomodulatory, and anti-carcinogenic properties [16, 17].

Propolis is a naturally occurring resinous mixture collected by honey bees from tree buds, sap, and other botanical sources. Due to its antiseptic and antimicrobial properties, propolis has long been utilized in folk medicine [18]. Propolis is a highly complex substance; thus, the chemical composition and biological properties of propolis obtained from different regions or countries can be very different [19, 20]. Importantly, different extraction processes can also yield different bioactive ingredients [21]. The composition and properties of propolis may also depend on the bee species that produced it due to their preferences for specific plants [22, 23].

In terms of the hypoglycaemic activity of propolis, several studies have shown that propolis from various countries, including Nigeria, Saudi Arabia, Brazil, and China, can lower glucose levels in diabetic rat models [7, 24, 25]. However, to our knowledge, there are very few studies of the biological and medicinal properties of Mexican propolis. Thus, the results presented here comprise the first evidence that a propolis of Mexican origin has a hypoglycaemic effect.

The Chihuahua propolis sample used in this study was collected from a hive of* Apis mellifera* honey bees. Treatment of diabetic mice with an ethanolic extract of this propolis for 15 days decreased blood glucose levels by approximately 40% compared with those levels in untreated diabetic mice. In addition, this same treatment prevented decreases in body weight of the mice. There are reports that the administration of propolis extracts from China and Brazil in rats with streptozotocin-induced diabetes leads to reduced blood glucose levels and prevents decreases in body weight [6, 25].

Insulin is a hormone secreted by *β*-cells of the islets of Langerhans in the pancreas. One function of insulin is to favour the incorporation of glucose into tissues, thereby decreasing blood glucose levels [26]. Therefore, once the Chihuahua propolis extract was shown to lower blood glucose levels, whether this effect was related to the production of insulin was evaluated. The insulin levels were determined in each of the experimental groups. Insulin was detected in diabetic mice treated with propolis (0.3 ± 0.02 ng/mL), whereas in the group diabetic mice insulin was not detected. The detection of insulin in propolis-treated diabetic mice suggests that the administration of Chihuahua propolis may have improved the secretion of insulin by the *β*-cells of the pancreatic islets, which may have been due to the possibility that propolis prevented further deterioration of the pancreatic islets. Some authors have suggested that propolis from Brazil shows activity in removing free radicals; together with the inhibition of IL-1*β* and nitric oxide synthase, these activities are probably the main factors for the protective effect of propolis [27]. In other reports with similar results, insulin levels (0.84 ± 0.3 ng/mL) can be detected after the administration of propolis from Saudi Arabia in experimental groups of diabetic rats [7].

Because insulin, a hormone that is only produced by the *β*-cells of the pancreatic islets, was detected, the presence of insulin in the pancreatic islets was evaluated in each of the experimental groups. The results showed that insulin was present in the islets of mice treated with propolis. There are few reports on propolis and insulin detection by immunohistochemistry. Nonetheless, some authors have reported that propolis from Nigeria possesses antioxidant compounds that could have indirect protective effects on pancreatic *β*-cells by stimulating the few surviving *β*-cells to secrete more insulin, thereby decreasing blood glucose levels [28].

Hyperglycaemia is an important factor responsible for the intense oxidative stress in diabetes, and the toxicity induced by glucose autoxidation is an important source of reactive oxygen species. There are several intra- and extracellular antioxidant defence mechanisms that counteract the destructive effects of free radicals by attenuating or inactivating these compounds. SOD, CAT, and GPx are antioxidant enzymes that play vital roles in preventing the exposure of cells to oxidative damage. SOD can reduce the superoxide radical in hydrogen peroxide (H_2_O_2_), whereas CAT and GPx reduce hydrogen peroxide to water and protect tissues against reactive hydroxyl radicals. In diabetes, high glucose levels can inactivate the antioxidant enzymes SOD, CAT, and GPx via glycation of these proteins, which produces oxidative stress that overcomes the antioxidant defence mechanisms of the body [7, 29]. Therefore, it is of great importance to identify compounds or natural products with good antioxidant capacities. In this study, the Chihuahua propolis extract was found to possess good antioxidant capacities (SA_50_ of 15.75 *μ*g/mL) based on Al-Fatimi et al.'s criteria for extracts with concentrations lower than 96.6 *μ*g/mL to be considered as having adequate antioxidant capacity [30]. In addition, the extract was found to consist of 31.4% phenols and 6.2% flavonoids. Propolis from other countries, such as China, Italy, Russia, and Brazil, has been reported to contain phenols and flavonoids as important parts of their chemical compositions, as these compounds have good antioxidant capacities. Some authors also mention that there is a direct correlation between the phenol and flavonoid contents and antioxidant capacity, as it has been shown that these compounds are able to eliminate the radicals that interfere with the normal cell metabolism, thereby protecting the cell membrane against lipid peroxidation [18, 19, 31]. In addition, in terms of antioxidant enzyme activity, in the propolis-treated diabetic, increases in the activities of each studied enzyme (SOD, CAT, and GPx) were observed compared with those in the untreated diabetic mice. Research carried out in diabetic rats has demonstrated that the administration of separate propolis extracts from China and Brazil increased the activity of SOD, CAT, and GPx enzymes [6, 25]. All of the effects described above are indispensable for counteracting the damage caused by free radicals that occur in diabetes-induced hyperglycaemia [32]. Thus, it can be suggested that treatment with propolis increases the activity of these enzymes in diabetic mice, and for the antioxidant defence system of the body to function well, there must be a decrease in the damage to different tissues, including the *β*-cells of the pancreatic islets, which are very susceptible to oxidative changes due to their low antioxidant capacity [6].

All of these results can be correlated with the data obtained from the chemical composition analysis, which showed that Chihuahua propolis is rich in flavonoids. Some of the compounds identified were naringin, pinocembrin, naringenin, kaempferol, quercetin, acacetin, luteolin, and chrysin. There are reports that flavonoids, such as flavones and flavonones, are among the main compounds in propolis [18]. Moreover, in studies performed using methanolic, aqueous, or ethanolic extracts of propolis from China, Italy, Russia, and Brazil, the main compounds identified included apigenin, luteolin, quercetin, kaempferol, galangin, pinobanksin, epicatechin, naringenin, pinocembrin, and chrysin [5, 31]. Flavonoids comprise a class of compounds that have received substantial attention for their potential roles as alternative treatments for complex diseases, such as diabetes, which implies alterations in multiple signalling pathways. Flavonoids are widely distributed in the plant kingdom and exhibit distinctive pharmacological properties [33]. As such, the results of the present study suggest that treatment with Chihuahua propolis, which contains several flavonoids, could affect different targets to lower blood glucose levels. Some authors have administered flavonoids, such as quercetin, naringin, or genistein, and have reported reduced blood glucose concentrations, detected insulin in serum or islets, and showed increased insulin release; these effects likely resulted from changes in Ca^2+^ metabolism, thus facilitating the hypoglycaemic effects of flavonoids [32–35]. Flavonoids have the ability to scavenge free radicals and chelate metals [36]. Given the hypothesized relation between diabetes and inflammation [37] and the potential for flavonoids to protect the body against free radicals and other prooxidative compounds [38], it is biologically plausible that the consumption of flavonoids or flavonoid-rich products may reduce the risk of diabetes complications [39, 40]. There is evidence that flavonoids such as naringin, a natural flavanone glycoside, has been widely used in traditional medicine. Naringin has been reported to possess antiapoptotic, antiosteoporosis, antiulcer, antioxidant, anti-inflammatory, and anticarcinogenic properties [41, 42]. Moreover, emerging data indicate that naringin is involved in ameliorating hyperglycaemia. Naringin possesses lipid‐lowering and insulin‐like properties that decrease insulin resistance, hyperglycaemia, and dyslipidaemia [43].

Another possible mechanism through which Chihuahua propolis might act is through its potent antioxidant capacity. Different studies have reported that the administration of quercetin in diabetic rats results in an increase in the activity of the antioxidant enzymes SOD, CAT, and GPx, which further protect the majority of Langerhans islet cells. It was thus concluded that quercetin treatment partially prevents *β*-cell degeneration, probably through antiapoptotic signals [34, 44].

Another possible effect of the Chihuahua propolis extract is the decreased absorption of glucose in the intestine via the inhibition of the enzymatic activity of *α*-glucosidase. Different* in vitro* and* in vivo* studies have reported that the administration of some flavonoids, such as acacetin, luteolin, chrysin, kaempferol, or naringenin, decreases glucose levels in diabetic rats and mice and shows inhibitory effects against the enzymatic activity of *α*-glucosidase [45–48]. Additionally, Chihuahua propolis might also have an effect on the reduction of serum glucose at the muscle level, as mentioned in some studies. Those researchers administered flavonoids such as quercetin or naringenin to isolated muscles and reported an increase in the uptake of glucose by skeletal muscle cells as well as the inhibition of the enzyme glycogen phosphorylase [49–51].

Chihuahua propolis might lower blood glucose levels via the activity of certain enzymes in the liver, as has been reported in several studies. In that previous research, the administration of flavonoids such as naringin, quercetin, or naringenin increased the concentration and activity of the hepatic enzyme glucokinase and markedly reduced the enzymatic activities of hepatic glucose-6-phosphatase and PEPCK. These findings suggested that the progression of hyperglycaemia is prevented partly by an increase in the hepatic concentration of glycolysis and/or by the reduction of hepatic gluconeogenesis [33, 49, 50, 52, 53].

This investigation demonstrated that propolis of Chihuahua possesses hypoglycaemic and antioxidant activities. These effects are directly related to its chemical composition, as most of the compounds found in propolis have been reported to have activities in terms of the different parameters evaluated in this work. However, more research is needed to increase the knowledge of all the biological effects of propolis, its possible adverse effects, and its mechanisms of action in order to promote the possible use of propolis in different therapeutic applications, including counteracting the various alterations caused by diabetes.

## Figures and Tables

**Table 1:** HPLC-DAD and HPLC-MS analysis of EPCh.

Name	Retention time (min)		*λ*_max_ (nm)	Parent ion *(m/z)* [M-H]^−^	Relative error (ppm)
	HPLC-DAD	HPLC-MS			
Naringin	5.71	17.116	214, 282	315.8400	2.98
Naringenin	7.88	24.071	290, 325 (sh)	271.0621	−3.18
Kaempferol	8.31	26.892	200, 266, 366	285.0412	−2.58
Quercetin	8.94	23.158	256, 372	301.0361	−2.48
Acacetin	9.40	32.32	210, 268, 324	283.0619	−2.32
Luteolin	10.05	22.51	252, 348	285.0774	−1.98
Pinocembrin	14.82	30.378	290	255.0672	−3.34
Chrysin	17.58	31.175	268, 314	253.0509	−1.18

## Supplementary Materials

Supplementary Materials Table 1: mice body weight after administration of the single oral dose of propolis at different concentrations (300, 600, 1200, 24000, and 5000 mg/Kg). Table 2: mice organ weight after administration of the single oral dose of propolis at different concentrations (300, 600, 1200, 24000, and 5000 mg/Kg). Table 3: hematological evaluation and biochemical analysis of the mice after administration of the single oral dose of propolis at different concentrations (300, 600, 1200, 24000, and 5000 mg/Kg).
