# Supplementary material for: Hypoglycaemic and Antioxidant Effects of Propolis of Chihuahua in a Model of Experimental Diabetes
Source: Evid Based Complement Alternat Med. 2018 Mar 11;2018:4360356. doi: 10.1155/2018/4360356 (PMC5866899; doi:10.1155/2018/4360356)
Supplement: Supplementary Materials — Table 1: mice body weight after administration of the single oral dose of propolis at different concentrations (300, 600, 1200, 24000, and 5000 mg/Kg). Table 2: mice organ weight after administration of the single oral dose of propolis at different concentrations (300, 600, 1200, 24000, and 5000 mg/Kg). Table 3: hematological evaluation and biochemical analysis of the mice after administration of the single oral dose of propolis at different concentrations (300, 600, 1200, 24000, and 5000 mg/Kg). [file 4360356.f1.pdf]

Evaluation of the acute toxicity of EPCh.

The acute toxicity of EPCh was evaluated by determining the lethal dose responsible for 50% death of the animals (LD_{50}). Mice were divided into seven groups with 6 animals each, and treated with a single dose of 300, 600, 1200, 24000 and 5000 mg of EPCh per kilogram orally. Vehicle group were treated with alcohol 25% (100μL). Control mice were treated with 100μL of filtered water only. The animals were constantly observed during the first 48 h. Before and at the end of the observation period, the animals were put under solid fasting for 12 h.

Toxicity and mortality signs were analyzed according to the methodology described by the OECD norm # 423 and were observed until 14 days after the treatment. During the observation period, the following parameters were measured at 3-day intervals: food and water consumption, and fatality. For each group, 150 g of food and 250 mL of filtered water were made available, which constituted their daily consumption.

After the observation period, the blood samples of the mice were obtained by cardiac puncture for hematological and biochemical analysis. Was determining total leukocyte count (TLC), hemoglobin, hematocrit, white blood corpuscles (RBCs), mean corpuscular volume (MCV), mean corpuscular hemoglobin (MCH), mean corpuscular hemoglobin concentration (MCHC), platelets, aspartate aminotransferase (AST), alanine aminotransferase (ALT), alkaline phosphatase (ALP), total protein, creatinine, blood urea nitrogen (BUN), calcium, phosphorous by means of automatic procedures.

Results

Regarding the study of acute toxicity performed in CD1 mice, the results obtained indicate that, regarding the general condition of the body and the weight of the animals, there were no significant differences between the groups with administration of EPCh in comparison with the control group (Table 1). In addition, mortality was not recorded of experimental animals after of propolis administration.

The weight of the organs (heart, lungs, kidneys, liver, spleen and stomach) that were obtained immediately after the sacrifice (day 14) of the mice with administration of EPCh did not show significant differences in comparison with the organs of the control group (Table 2).

The administration of EPCh did not alter the hematological and biochemical parameters of any of the animals when compared with the control group (Table 3).

Table 1. Mice body weight after administration of the single oral dose of propolis at different concentrations (300, 600, 1200, 24000 and 5000 mg/Kg).

Concentration (mg/Kg)	Mortality	Signs of toxicity	Survival (%)	Body weight (g)			
				Day 0	Day 1	Day 7	Day 14
Control	0/3	0/3	100	32.33±1.52	32.33±1.04	35±1.41	38.83±1.75
Vehicle (OH-25%)	0/3	0/3	100	31.67±1.39	31.6±0.9	34.4±1.9	37.3±1.3
300	0/3	0/3	100	32.17±1.72	32.86±1.21	35.7±1.5	38.26±0.95
600	0/3	0/3	100	31.13±0.98	31.46±0.57	34.4±1.02	37.9±1.22
1200	0/3	0/3	100	33.67±1.16	33.76±1.43	36.89±1.34	39.9±0.46
2400	0/3	0/3	100	31.63±0.85	32±0.91	36.17±1.75	38.75±0.95
5000	0/3	0/3	100	33.17±1.04	33.33±1.04	36.33±0.57	38.33±1.52

Table 2. Mice organ weight after administration of the single oral dose of propolis at different concentrations (300, 600, 1200, 24000 and 5000 mg/Kg).

Concentration (mg/Kg)	Weight of organs at day 14 (g)					
	Heart	Lungs	Kidneys	Spleen	Liver	Stomach
Control	0.170±0.01	0.231±0.02	0.532±0.02	0.135±0.01	1.924±0.01	0.308±0.008
Vehicle (OH-25%)	0.173±0.07	0.220±0.62	0.563±0.04	0.124±0.03	1.846±0.32	0.329±0.1
300	0.172±0.06	0.219±0.36	0.548±0.077	0.118±0.004	1.821±0.13	0.346±0.04
600	0.175±0.39	0.233±0.05	0.495±0.035	0.117±0.006	1.912±0.16	0.317±0.05
1200	0.167±0.95	0.227±0.24	0.576±0.08	0.134±0.02	1.860±0.24	0.343±0.03
2400	0.173±0.006	0.248±0.04	0.573±0.005	0.127±0.005	1.858±0.10	0.333±0.02
5000	0.161±0.002	0.217±0.01	0.489±0.02	0.112±0.008	1.837±0.07	0.355±0.02

Values are expressed as mean± S.D, n=6

Table 3. Hematological evaluation and biochemical analysis of the mice after administration of the single oral dose of propolis at different concentrations (300, 600, 1200, 24000 and 5000 mg/Kg).

Hematological parameters	Normal values	Units	Experimental groups						
			Control	Vehicle (OH-25%)	Propolis concentrations (mg/Kg)				
					300	600	1200	2400	5000
TLC	3-14.2	$1x10^3$ células/μL	10.46±3.57	8.86±1.21	9.1±0.17	11.9±0.5	10.5±0.6	8±1.41	9.83±0.75
Hemoglobin	10.9-16.3	g/dL	11.2±0.85	12.4±1.02	13.33±0.52	11.33±1.04	10.63±0.85	12±0.91	11.17±1.05
Hematocrit	38.5-45.1	%	39.7±0.5	41.3±1.06	43.75±0.95	39.33±0.57	40.33±1.04	42.33±1.52	40.17±1.04
MCV	48-56	fL ($1x10^{-15}$/L)	51.7±2.7	53.6±1.9	49.3±1.04	53.2±1.02	52.8±1.07	49.5±0.5	50±1.08
MCH	11.9-19	pg	13.8±0.78	16.3±1.3					
MCHC	25.9-35.1	g/dL	30.9±0.46	31.4±0.72	31.7±0.55	29.43±0.63	30.5±0.78	28.3±0.92	31.16±0.95
Platelets	1084-1992	$1x10^3$ platelets/μL	1530.3±15.07	1270±17.69	1380.3±19	1460±8.46	1370.5±8.2	1510.7±10.8	1470.5±19.2
Biochemical parameters									
AST	69-191	U/L	80.89±2.34	85.26±6.95	83.7±4.07	85.8±3.10	86±5.24	82.1±2.13	84.6±3.32
ALT	26-120	U/L	31.4±2.9	42.3±0.78					
ALP	44-118	U/L	49.46±0.43	51.4±0.9	53±1.05	48.9±2.02	50.3±1.8	49.2±1.1	54.6±2.04
Total proteins	43-64	g/dL	45.46±0.43	46.4±0.9	49.91±0.16	44.24±0.81	48.31±0.42	45.19±0.36	47.20±0.62
Creatinin	0.5-0.8	mg/dL	0.67±0.16	0.7±0.16	0.61±0.13	0.78±0.04	0.59±0.06	0.74±0.12	0.67±0.15
BUN	19-34	mg/dL	24.13±2.98	22.17±1.72	23.3±1.05	22.7±2.24	24.8±1.04	21.7±1.01	23.5±2.01
Calcium	9-12	mmol/L	10.9±0.22	10.22±0.78	11.3±0.66	11.6±0.52	10.7±0.39	11.2±0.46	10±0.7
Phosphorus	6-13	mmol/L	7.42±0.18	8.67±0.39	10.21±0.36	11.2±0.28	9.12±0.16	10.02±0.62	8.37±0.22

Values are expressed as mean± S.D, n=6
